# Value of contrast-enhanced ultrasound for preoperative assessment of liver reserve function in patients with liver tumors

**DOI:** 10.1371/journal.pone.0222514

**Published:** 2019-09-17

**Authors:** Huiming Yi, Baohuan Cai, Xi Ai, Ruobing Liu, Kaiyan Li, Wei Zhang

**Affiliations:** 1 Department of Medical Ultrasound, Tongji Hospital, Tongji Medical College, Huazhong University of Science and Technology, Wuhan City, Hubei Province, China; 2 Department of Pediatrics, Tongji Hospital, Tongji Medical College, Huazhong University of Science and Technology, Wuhan City, Hubei Province, China; 3 Department of Hepatic Surgery, Tongji Hospital, Tongji Medical College, Huazhong University of Science and Technology, Wuhan City, Hubei Province, China; Chinese Academy of Medical Sciences and Peking Union Medical College, CHINA

## Abstract

This study aimed to investigate the value of contrast-enhanced ultrasound (CEUS) for preoperative assessment of liver reserve function in patients with liver tumors. The indocyanine green (ICG) clearance tests and CEUS examinations of 45 noncirrhotic patients with liver tumors were performed prior to liver resection. Parameters time to peak (TtoPk), arrival time (Atm) as well as perfusion parameters A, k and A x k were generated from time-intensity curve (TIC) of CEUS. The correlation analyses of the ICG clearance per unit time (ICGK) and the retention rate at 15 min (ICGR15) with TtoPk, Atm, A, k and A x k were performed, and the diagnostic ability as well as optimal cut-off values of TtoPk and Atm for differentiating patients with ICGR15>10% from ICGR15<10% were analyzed. There were significant correlations of ICGK with TtoPk and Atm, and the correlation coefficients were 0.363 (p = 0.014) and -0.482 (p = 0.001), respectively. Significant correlations of ICGR15 with TtoPk and Atm were revealed, and the correlation coefficients were -0.416 (p = 0.004) and 0.303 (p = 0.043), respectively. No correlation of ICGK or ICGR15 with A, k and A x k was found in this study. There were significant differences in TtoPk and Atm between patients with ICGR15>10% and ICGR15<10% (p = 0.028 and p = 0.026, respectively). TtoPk and Atm both had good diagnostic abilities in diagnosing patients with ICGR15>10% verusus ICGR15<10% (AUROC = 0.711 and 0.721, respectively). For ICGR15>10% vs ICGR15, the optimal cut-off values of TtoPk and Atm were 13.307 s and 11.007 s, respectively, while the sensitivity and specificity were 75.0% and 72.7%, 60.6% and 75.0%, respectively. This study revealed that CEUS has the potential to be a new method to evaluate the liver reserve function of patients. With the optimal cut-off values of TtoPk and Atm, qualitative assessment of patients with ICGR15>10% could be more easily achieved by CEUS with good diagnostic abilities.

## Introduction

The indocyanine green (ICG) clearance test is a useful method for comprehensive evaluation of liver function in clinical practice and the ICG clearance per unit time (ICGK) and the retention rate at 15 min (ICGR15) post injection represent indicators with a high sensitivity and specificity for evaluating liver reserve function[1, 2].

Studies indicated that results of preoperative ICG clearance test were closely related to postoperative outcome after hepatectomy[3, 4]. Therefore, the ICG clearance test has been regarded as the most frequently used as well as the most effective method for the preoperative assessment of the hepatic reserve function and the operation risk before hepatectomy[1, 5, 6].

Contrast-enhanced ultrasonography (CEUS) can provide real-time dynamic imaging of tissues, from which time-intensity curve (TIC) and quantitative perfusion parameters can be derived[7–9]. At present, with the of advantages of low cost, portability and non-ionizing radiation, CEUS has been considered as the main imaging technique for the exploration and localization of liver tumors, interventional ablation guidance and therapy evaluation[10, 11]^.^ However, the relationship between the CEUS and the liver reserve function has not been established yet. In this study, the correlation analyses between parameters of TIC from CEUS and the ICG-R15 and ICGK of patients prior to liver resection were performed in order to investigate the value of CEUS for preoperative assessment of liver reserve function in patients with liver tumors.

## Materials and methods

### Patients

45 noncirrhotic patients with liver tumors from Tongji Hospital between July 2017 and July 2018 were enrolled in this study. The protocols of this study were approved by the Ethics Committee of the Tongji Hospital, Tongji Medical College, Huazhong University of Science and Technology and written informed consents were obtained from all participating patients. All data were anonymized during analysis.

### Contrast-enhanced ultrasound

CEUS examinations were performed by Logiq E9 doppler ultrasonic diagnosis apparatus (General Electric Company, Fairfield, Connecticut, USA) equipped with a 4 MHz harmonic-imaging transducer. SonoVue (Bracco Imaging, Milan, Italy), a second generation of contrast agent composed of microbubbles of sulphur hexafluoride, was resolved in normal saline at a ratio of 1:5 ml and agitated for complete dissolution. Each patient underwent bolus injection of 2.4 ml of SonoVue solution followed by 5 ml flush of saline in bolus via cubital vein. A dynamic image of CEUS was recorded until the contrast agent diminished following contrast injection. Region of interest (ROI) was placed in the liver parenchyma of the left lobe to develop time-intensity curve(TIC) generated by software automatically, by which time to peak (TtoPk), arrival time (Atm) and perfusion parameters were calculated from curve fitting formula F(t) = A(1-exp[-kt])+B): where A is the plateau value as an estimate of the regional blood volume, k is the replenishment rate as an estimate of microbubble velocity, and A x k is an estimate of flow, B is the baseline and t is the time[12]

### The ICG clearance test

The ICG clearance tests were performed using DDG-3300K analyzer (Pulse Dye Densito-Graph analyzer, Nihon Kohden Corp., Japan) within 12 hours before or after CEUS examinations prior to liver resection. Patient lied down with nose connected to the nasal probe of DDG-3300K analyzer, then the ICG solution (0.5 mg per kilogram bodyweight) was injected through cubital vein within 5 seconds, the ICGK and ICGR15 values were calculated automatically by the analyzer.

### Statistical analysis

Statistical analyses were performed using SPSS 19 software (SPSS Inc., Chicago, Illinois, USA) and a p-value < 0.05 was regarded as statistically significant difference. Continuous variables were presented as mean ± standard deviation (SD) and analyzed using Student’s t-test. The correlation analyses of ICG-R15 and ICGK with parameters of TIC curve from CEUS were performed using the Pearson rectilinear correlation analysis and linear regression analysis. The diagnostic abilities of TtoPk and Atm to differentiate patients with ICGR15>10% from ICGR15<10% were evaluated using receiver operating characteristic curve (ROC) analysis. The maximum Youden Index defined as sensitivity+specificity−1 was used to determine the optimal cut-off values[13]. Diagnostic ability was classified as low (the area under the ROC curve (AUROC) = 0.50–0.70), moderate (AUROC = 0.70–0.90), or high (AUROC = 0.90–1.0) [14].

## Results

### Clinical features

A total of 45 patients with tumors in the right lobe of the liver were enrolled for this study. Of these patients, 39 were males and 6 were females with median age of 54.7 years (36–72 years). There were 33 patients with ICGR15<10%, and 12 patients with ICGR15>10%. All patients included were Child-Pugh class A and noncirrhotic. The characteristics, ICGR15, ICGK and parameters of TIC from CEUS of patients included were presented in Table 1.

**Table 1:** Characteristics, ICGR15, ICGK and parameters of TIC from CEUS of patients.

	Total (n = 45)	ICGR15<10% (n = 33)	ICGR15>10% (n = 12)	ICGR15<10% vs ICGR15>10%
Age(years)	54.7±10.8	53.1±11.1	59.0±8.4	p = 0.78
Height(cm)	169.0±6.3	170.0±6.8	169.6±4.7	p = 0.84
Weight(kg)	63.5±10.9	63.3±10.8	64.1±11.1	p = 0.110
ALT(U/L)	30.0±16.3	28.5±16.0	34.2±16.6	p = 0.318
AST(U/L)	29.7±13.6	25.7±11.0	40.5±14.2	p = 0.001
TBil(μmol/L)	13.7±6.3	12.4±4.9	17.4±8.1	p = 0.020
DBil(μmol/L)	4.8±2.9	3.9±1.9	7.0±3.8	p = 0.001
Albumin (g/L)	45.6±3.7	46.1±3.3	44.3±4.2	p = 0.161
GGT (U/L)	65.7±52.3	59.1±51.6	84.0±49.8	p = 0.165
ALP (U/L)	84.0±27.8	80.5±27.4	93.6±26.7	p = 0.172
ICGK(/min)	0.209±0.076	0.246±0.049	0.108±0.036	
ICGR15(%)	8.96±11.40	3.17±2.22	24.88±11.14	
TtoPk(s)	12.054±4.747	12.992±4.837	9.474±3.328	p = 0.028
Atm(s)	12.808±3.873	12.035±3.476	14.934±4.106	p = 0.026
A	23.551±4.933	23.637±5.036	23.314±4.865	p = 0.852
k	0.319±0.145	0.319±0.151	0.321±0.124	p = 0.960
A x k	7.580±4.007	7.632±4.252	7.437±3.232	p = 0.888

ALT, alanine aminotransferase; AST, aspartate aminotransferase; TBil, total bilirubin; DBil, direct bilirubin; GGT, Gamma-glutamyltransferase; ALP, alkaline phosphatase; ICG, indocyanine green

ICGK, ICG clearance per unit time; ICGR15, retention rate of ICG at 15 min

CEUS, contras-enhanced ultrasound; TIC, time-intensity curve; TtoPk, time to peak; Atm, arrival time.

### The correlation analyses of ICGK and ICG-R15 with parameters of TIC from CEUS

**Fig 1:**
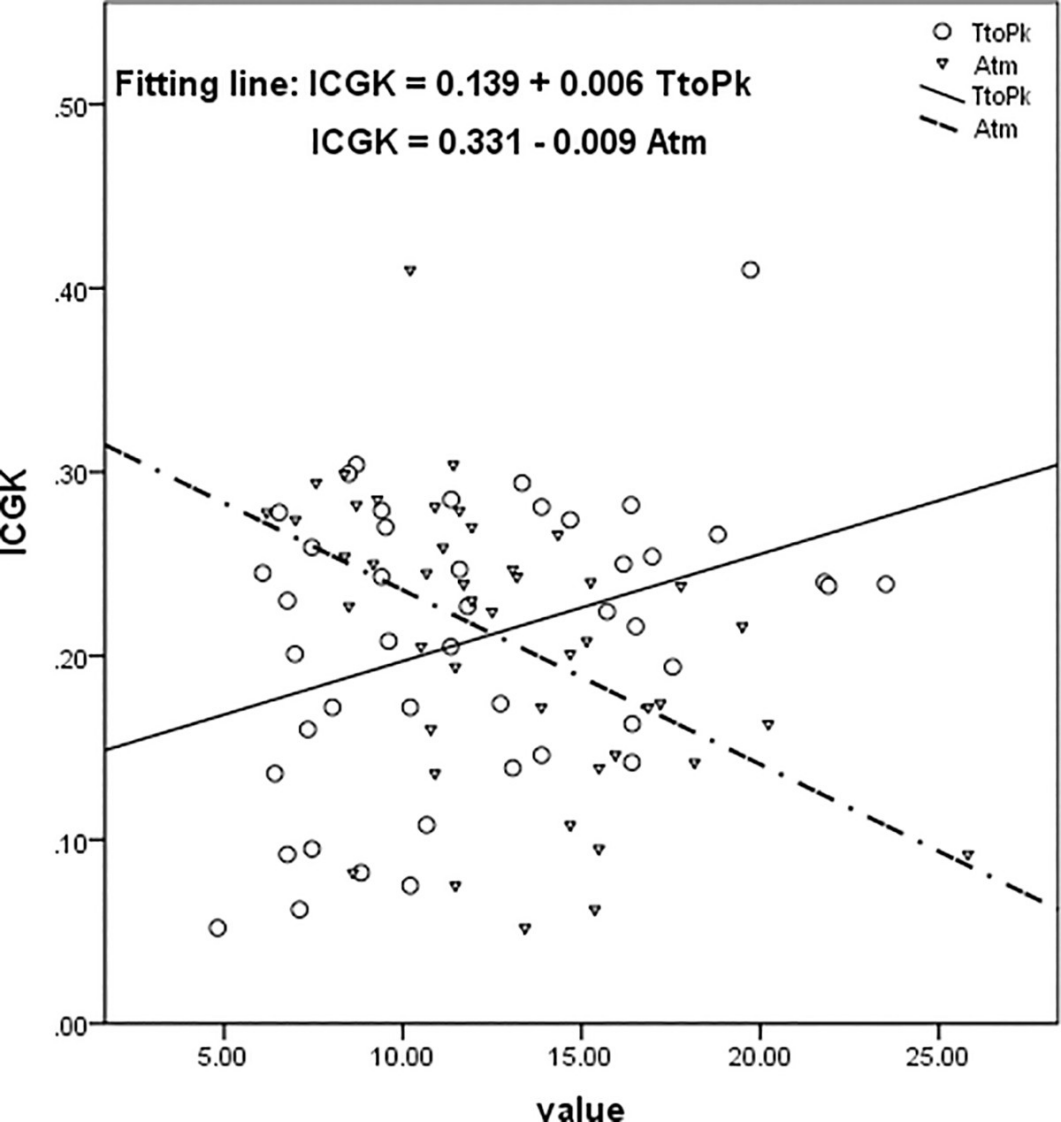
Results of correlation analyses of ICGK with TtoPk and Atm. ICGK is positively correlated with TtoPk, while negatively correlated to Atm. ICGK, ICG clearance per unit time; ICGR15, retention rate of ICG at 15 min; TtoPk, time to peak; Atm, arrival time.

**Fig 2:**
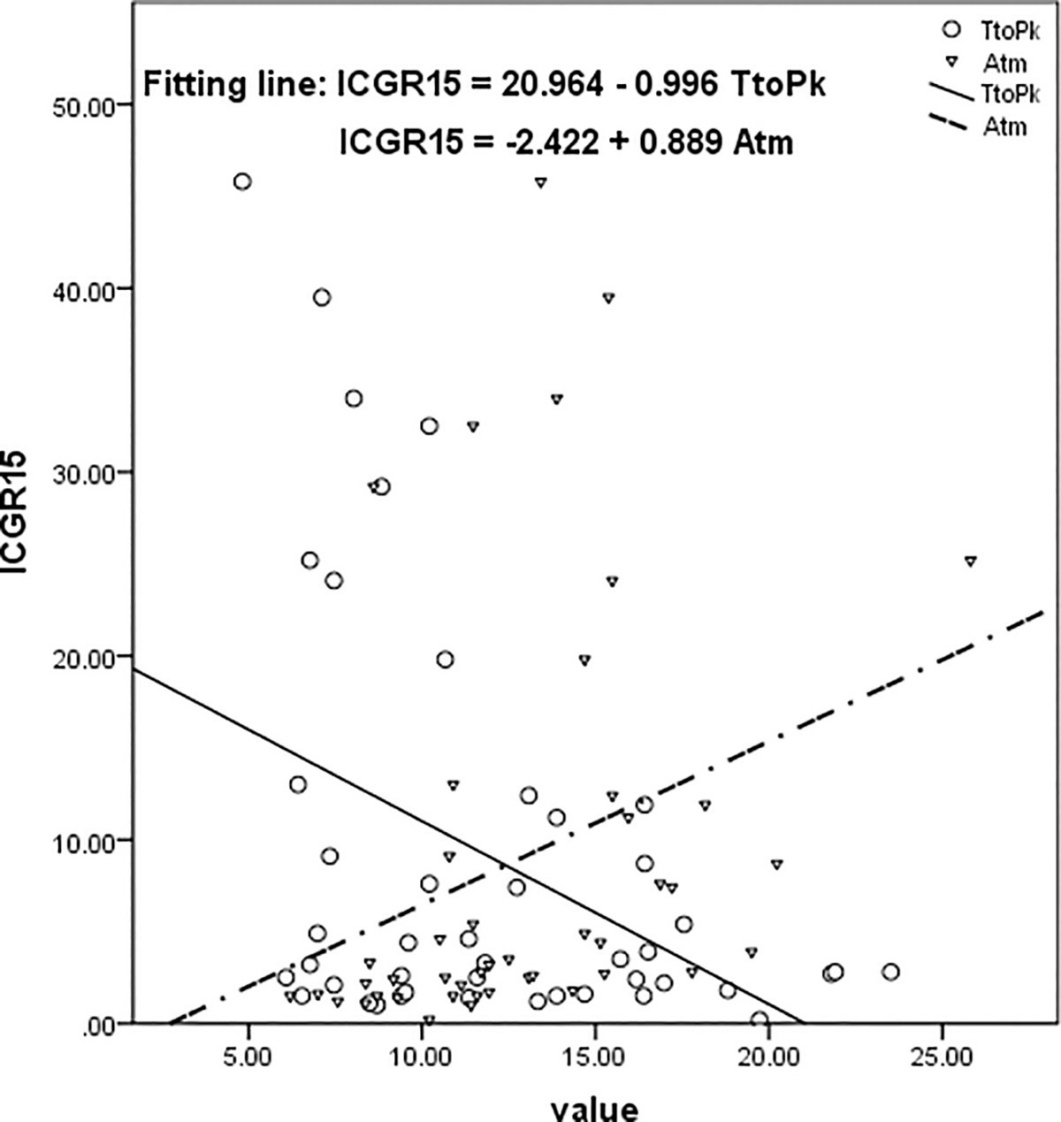
Results of correlation analyses of ICGK with TtoPk and Atm. ICGR15 is negatively correlated with TtoPk, while positively correlated to Atm. ICGK, ICG clearance per unit time; ICGR15, retention rate of ICG at 15 min; TtoPk, time to peak; Atm, arrival time.

Results of the correlation between ICGK and ICG-R15 and parameters of TIC from CEUS were shown in Table 2. There were significant correlations of ICGK with TtoPk and Atm, and the correlation coefficients were 0.363 (p = 0.014) and -0.482 (p = 0.001), respectively (Fig 1). Significant correlations of ICGR15 with TtoPk and Atm were revealed, and the correlation coefficients were -0.416 (p = 0.004) and 0.303 (p = 0.043), respectively (Fig 2). No correlation of ICGK or ICGR15 with A, k and A x k was found in this study.

**Table 2:** The correlation coefficients of ICGK and ICGR15 with parameters of TIC from CEUS.

	TtoPk	Atm	A	k	A x k
ICGK	0.363(p = 0.014)	-0.482(p = 0.001)	0.151(p = 0.321)	0.012(p = 0.939)	0.078(p = 0.609)
ICGR15	-0.416(p = 0.004)	0.303(p = 0.043)	0.076(p = 0.619)	-0.021(p = 0.892)	-0.012(p = 0.936)

TtoPk, time to peak; Atm, arrival time.

### ROC analyses of TtoPk and Atm in differentiating patients with ICGR15>10% from ICGR15<10%

**Fig 3:**
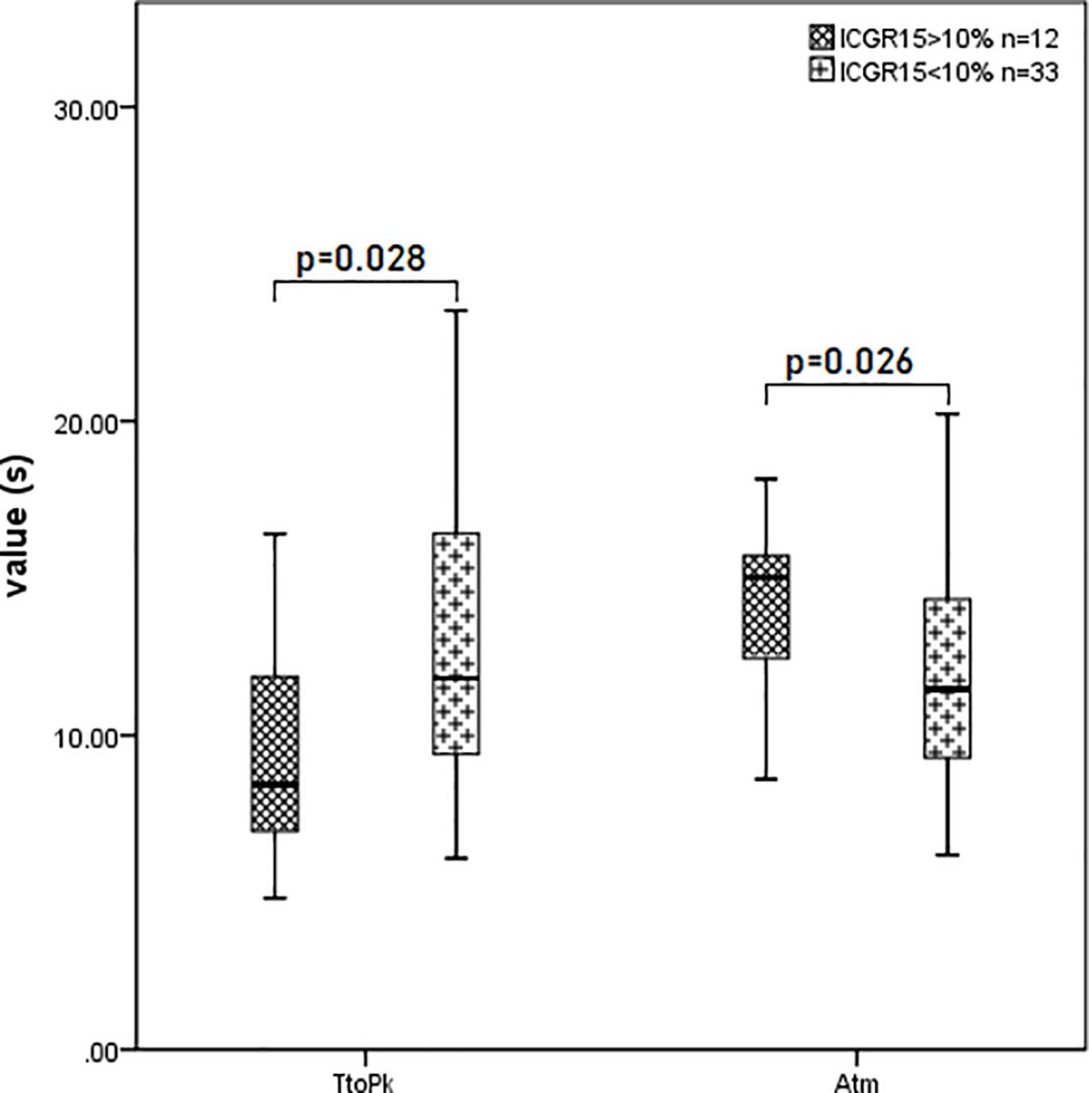
Relationship between patients with ICGR15>10% and ICGR15<10% investigated by TtoPk and Atm. TtoPk decreased in patients with ICGR15>10%, while Atm increased in patients with ICGR15>10%. TtoPk, time to peak; Atm, arrival time.

**Fig 4:**
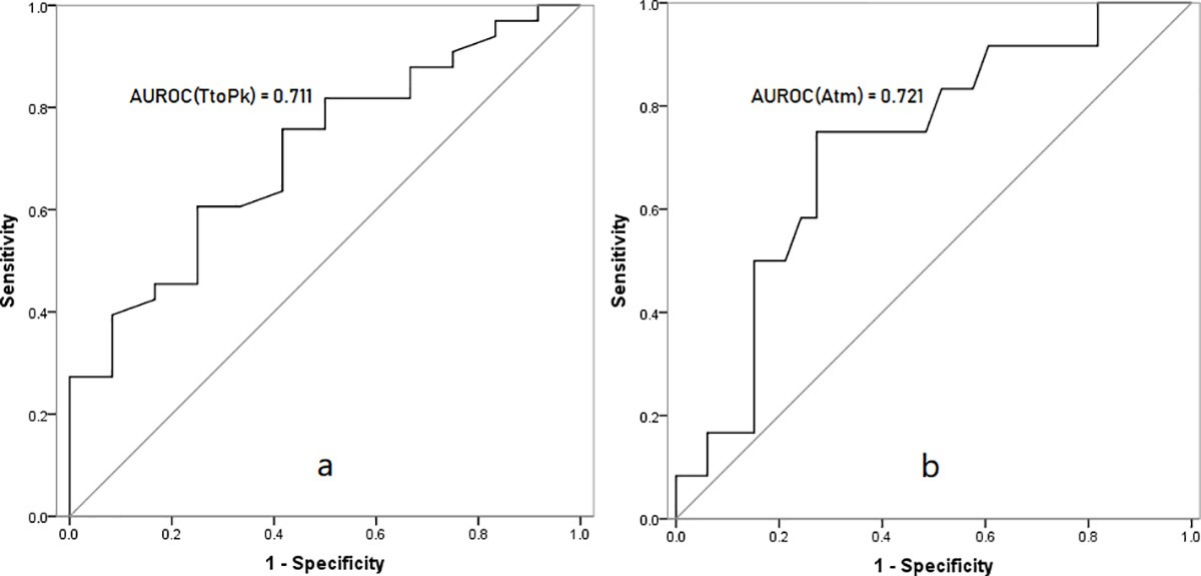
**ROC analyses of TtoPk (a) and Atm (b) for diagnosing ICGR15>10% versus ICGR15<10%.** TtoPk, time to peak; Atm, arrival time; AUROC, area under ROC.

There were significant differences in TtoPk and Atm between patients with ICGR15>10% and ICGR15<10% (p = 0.028 and p = 0.026, respectively). (Fig 3 and Table 1). The ROC curves of TtoPk and Atm for differentiating patients with ICGR15>10% from ICGR15<10% were shown in Fig 4. The AUROC values of TtoPk and Atm were 0.711 and 0.721, respectively, corresponding to moderate diagnostic ability.

Using 13.307 s as the optimal cut-off value of TtoPk according to the Youden index, the sensitivity and specificity were 75.0% and 72.7%, respectively. Using 11.007 s as the optimal cut-off value of Atm according to the Youden index, the sensitivity and specificity were 60.6% and 75.0%, respectively. (Table 3)

**Table 3:** Optimal cut-off values of TtoPk and Atm for differentiating patients with ICGR15>10% from ICGR15<10%.

	Optimal cut-off value(s)	Sensitivity	Specificity	Youden Index
TtoPk	13.307	75.00%	72.70%	0.477
Atm	11.007	60.60%	75.00%	0.356

TtoPk, time to peak; Atm, arrival time

## Discussion

Liver resection is an effective surgical treatment for a broad variety of benign and malignant hepatic tumors, post-hepatectomy liver failure (PHLF) remained the most serious complication and the major cause of mortality after liver resections, particularly in patients with suboptimal liver function due to parenchymal liver disease such as cirrhosis or steatosis[15, 16]. Therefore, it is important to estimate liver reserve function before planning partial resection of the liver in order to predict function of the remnant liver[17].

ICG is a water-soluble tricarboxylic acid dye, exclusively taken up by hepatocytes and excreted unchanged into the bile following intravenous injection without extrahepatic metabolism and excretion[18]. The ICG clearance test is a well-established method in evaluating liver reserve function, and with the development of noninvasive pulse spectrophotometers, the ICG clearance test has been routinely used for many years in patients before hepatectomy, ICG-K and ICGR15 measurements have also long been part of standard clinical evaluation of liver function reserve for patients prior to liver resection[19, 20]. Clearance of serum ICG by the liver is regulated by the function of hepatocytes and hepatic blood flow. Moreover, it has been reported that after an intravenous bolus injection of ICG, the uptake of ICG from plasma to hepatocytes was affected by hepatic microcirculatory condition, and the removal of ICG from the hepatocytes by cytoplasmic transport and biliary excretion referred the parenchymal liver function[21, 22].

CEUS with microbubble contrast agents has been a valuable imaging technique used to estimate the tissue hemodynamics. With the time-intensity curve (TIC) and the related parameters generated from CEUS based on signal intensity of contrast agent in the region of interest, tissue perfusion can be evaluated quantitatively[23].

In the present study, the correlation analyses of ICG-R15 and ICGK with parameters (TtoPk, Atm, A, k, A x k) of TIC from CEUS were performed to explore the value of CEUS in assessment of liver reserve function in noncirrhotic patients with liver tumors. Results of the present study revealed that ICGR15 is negatively correlated with TtoPk and positively correlated to Atm, while ICGK is positively correlated with TtoPk, and negatively correlated to Atm. The significant correlations of ICG-R15 and ICGK with TtoPk and Atm demonstrated that CEUS has the potential to be a new method to evaluate the liver reserve function of patients.

Furthermore, this study explored the feasibility and accuracy of the parameters TtoPk and Atm to differentiate patients with ICGR15>10% from ICGR15<10%. Using ROC analyses, we found that TtoPk and Atm both had good diagnostic abilities in diagnosing patients with ICGR15>10% verusus ICGR15<10% (AUROC = 0.711 and 0.721, respectively). This study also revealed that for ICGR15>10% vs ICGR15, the optimal cut-off values of TtoPk and Atm were 13.307 s and 11.007 s, respectively, and the sensitivity and specificity were 75.0% and 72.7%, 60.6% and 75.0%, respectively. According to the correlations of TtoPk and Atm with liver reserve function as well as the optimal cut-off values of TtoPk and Atm, qualitative assessment of liver reserve function could be more easily achieved.

This study has several limitations. The results of the present study are only applicable to Child-Pugh class A and noncirrhotic patients. In addition, due to the limited number of patients and the single-center design, the results are preliminary and a further large-scale multi-center study is needed.

## Conclusion

This study revealed significant correlation of ICG-R15 and ICGK with TtoPk and Atm, which demonstrated that CEUS has the potential to be a new method to evaluate the liver reserve function of patients. With the optimal cut-off values of TtoPk and Atm, qualitative assessment of patients with ICGR15>10% could be more easily achieved by CEUS with good diagnostic abilities.

## References

[1] Sheng QS, Lang R, He Q, Yang YJ, Zhao DF, Chen DZ. Indocyanine green clearance test and model for end-stage liver disease score of patients with liver cirrhosis. Hepatobiliary Pancreat Dis Int. 2009;8(1):46–9. 19208514

[2] Gupta S, Chawla Y, Kaur J, Saxena R, Duseja A, Dhiman RK, et al Indocyanine green clearance test (using spectrophotometry) and its correlation with model for end stage liver disease (MELD) score in Indian patients with cirrhosis of liver. Trop Gastroenterol. 2012;33(2):129–34. 23025060 10.7869/tg.2012.30

[3] Haegele S, Reiter S, Wanek D, Offensperger F, Pereyra D, Stremitzer S, et al Perioperative non-invasive indocyanine green-clearance testing to predict postoperative outcome after liver resection. PloS one. 2016;11(11):e0165481 10.1371/journal.pone.0165481 27812143 PMC5094749

[4] Wang YY, Zhao XH, Ma L, Ye JZ, Wu FX, Tang J, et al Comparison of the ability of Child-Pugh score, MELD score, and ICG-R15 to assess preoperative hepatic functional reserve in patients with hepatocellular carcinoma. Journal of surgical oncology. 2018;118(3):440–45. 10.1002/jso.25184 30259515

[5] Wang L, Xie L, Zhang N, Zhu W, Zhou J, Pan Q, et al Predictive Value of Intraoperative Indocyanine Green Clearance Measurement on Postoperative Liver Function After Anatomic Major Liver Resection. Journal of Gastrointestinal Surgery. 2019;13:1–10. 10.1007/s11605-019-04262-5 31197694

[6] Seyama Y, Kokudo N. Assessment of liver function for safe hepatic resection. Hepatol Res. 2009;39(2):107–16. 10.1111/j.1872-034X.2008.00441.x 19208031

[7] Wildner D, Schellhaas B, Strack D, Goertz RS, Pfeifer L, Fiessler C, et al Differentiation of malignant liver tumors by software-based perfusion quantification with dynamic contrast-enhanced ultrasound (DCEUS). Clinical hemorheology and microcirculation. 2019;71(1):39–51. 10.3233/CH-180378 29865043

[8] Cosgrove D, Harvey C. Clinical uses of microbubbles in diagnosis and treatment. Med Biol Eng Comput. 2009;47(8):813–26. 10.1007/s11517-009-0434-3 19205774

[9] Quaia E. Assessment of tissue perfusion by contrast-enhanced ultrasound. Eur Radiol. 2011;21(3):604–15. 10.1007/s00330-010-1965-6 20927527

[10] Wang Z, Dabrosin C, Yin X, Fuster MM, Arreola A, Rathmell WK, et al Broad targeting of angiogenesis for cancer prevention and therapy. Semin Cancer Biol. 2015;35 Suppl:S224–S43. 25600295 10.1016/j.semcancer.2015.01.001 PMC4737670

[11] Zhan Y, Zhou FB, Yu XL, Luo F, Liu FY, Liang P, et al Quantitative dynamic contrast-enhanced ultrasound may help predict the outcome of hepatocellular carcinoma after microwave ablation. Int J Hyperther. 2019;35(1):105–11. 10.1080/02656736.2018.1483533 30300039

[12] Wu HP, Patel RB, Zheng YY, Solorio L, Krupka TM, Ziats NP, et al Differentiation of Benign Periablational Enhancement from Residual Tumor Following Radio-Frequency Ablation Using Contrast-Enhanced Ultrasonography in a Rat Subcutaneous Colon Cancer Model. Ultrasound in Medicine and Biology. 2012;38(3):443–53. 10.1016/j.ultrasmedbio.2011.12.008 22266229 PMC3280615

[13] Youden WJ. Index for Rating Diagnostic Tests. Cancer. 1950;3(1):32–5. 10.1002/1097-0142(1950)3:1<32::aid-cncr2820030106>3.0.co;2-3 15405679

[14] Swets JA. Measuring the Accuracy of Diagnostic Systems. Science. 1988;240(4857):1285–93. 10.1126/science.3287615 3287615

[15] Takano S, Oishi H, Kono S, Kawakami S, Nakamura M, Kubota N, et al Retrospective analysis of type of hepatic resection for hepatocellular carcinoma. Brit J Surg. 2000;87(1):65–70. 10.1046/j.1365-2168.2000.01308.x 10606913

[16] Erdogan D, Heijnen BH, Bennink RJ, Kok M, Dinant S, Straatsburg IH, et al Preoperative assessment of liver function: a comparison of 99mTc-Mebrofenin scintigraphy with indocyanine green clearance test. Liver Int. 2004;24(2):117–23. 10.1111/j.1478-3231.2004.0901.x 15078475

[17] Ibis C, Albayrak D, Sahiner T, Soytas Y, Gurtekin B, Sivrikoz N. Value of Preoperative Indocyanine Green Clearance Test for Predicting Post-Hepatectomy Liver Failure in Noncirrhotic Patients. Med Sci Monitor. 2017;23:4973–80. 10.12659/MSM.907306 PMC5657461 29042529

[18] El-Desoky A, Seifalian AM, Cope M, Delpy DT, Davidson BR. Experimental study of liver dysfunction evaluated by direct indocyanine green clearance using near infrared spectroscopy. Brit J Surg. 1999;86(8):1005–11. 10.1046/j.1365-2168.1999.01186.x 10460634

[19] Kim HJ, Kim CY, Park EK, Hur YH, Koh YS, Kim HJ, et al Volumetric analysis and indocyanine green retention rate at 15 min as predictors of post-hepatectomy liver failure. Hpb. 2015;17(2):159–67. 10.1111/hpb.12295 24964188 PMC4299390

[20] De Gasperi A, Mazza E, Prosperi M. Indocyanine green kinetics to assess liver function: Ready for a clinical dynamic assessment in major liver surgery? World J Hepatol. 2016;8(7):355–67. 10.4254/wjh.v8.i7.355 26981173 PMC4779164

[21] Shinohara H, Tanaka A, Kitai T, Yanabu N, Inomoto T, Satoh S, et al Direct measurement of hepatic indocyanine green clearance with near-infrared spectroscopy: Separate evaluation of uptake and removal. Hepatology. 1996;23(1):137–44. 10.1053/jhep.1996.v23.pm0008550033 8550033

[22] Chijiiwa K, Watanabe M, Nakano K, Noshiro H, Tanaka M. Biliary indocyanine green excretion as a predictor of hepatic adenosine triphosphate levels in patients with obstructive jaundice. Am J Surg. 2000;179(2):161–6. 10.1016/s0002-9610(00)00274-9 10773154

[23] Ohno N, Miyati T, Yamashita M, Narikawa M. Quantitative Assessment of Tissue Perfusion in Hepatocellular Carcinoma Using Perflubutane Dynamic Contrast-Enhanced Ultrasonography: A Preliminary Study. Diagnostics. 2015;5(2):210–8. 10.3390/diagnostics5020210 26854150 PMC4665596

